# Distinguishing Iron Deficiency Anemia From Beta-Thalassemia Trait: Comparative Analysis of CRUISE Index and Other Traditional Diagnostic Indices

**DOI:** 10.7759/cureus.64048

**Published:** 2024-07-07

**Authors:** Agam Hans, Charu B Atreja, Neha Batra

**Affiliations:** 1 Pathology, Maharishi Markandeshwar Institute of Medical Sciences and Research, Ambala, IND; 2 Pathology, Punjab Institute of Medical Sciences, Jalandhar, IND

**Keywords:** microcytic hypochromic anemia, cruise index, mentzer index, beta thalassemia trait, iron deficiency anemia (ida)

## Abstract

Introduction

Iron deficiency anemia and beta-thalassemia trait are two common and important differentials of microcytic hypochromic anemia. Various discrimination indices using two or more common complete blood cell count (CBC) parameters have been used to distinguish between the two since 1973. Recently, a new discriminant index, the CRUISE index, was proposed in the year 2019. The efficacy of various older indices along with the CRUISE index was evaluated for patients in our geographical area.

Materials and method

Ours was a laboratory-based, cross-sectional study where 100 patients, based on inclusion and exclusion criteria, with microcytic hypochromic anemia were evaluated for CBC parameters along with serum ferritin and hemoglobin-high performance liquid chromatography (Hb HPLC). A total of eight discrimination indices namely, Mentzer, Srivastava, Shine & Lal, Green & King, RDWI, England & Fraser, Kerman I and CRUISE index were used and evaluated for their diagnostic efficacy using different statistical parameters. ROC curves were obtained and a new cut-off value was proposed for our population. Data was analysed using Microsoft Excel (Microsoft^®^ Corp., Redmond, WA, USA) and SPSS v29.0.2.0 (20) (IBM Corp., Armonk, NY, USA).

Results

Out of the total 100 cases, 39 were beta-thalassemia trait and 61 were iron deficiency anemia cases. The average age was 36.7 (±12.7 SD) years. Among the 73 females, 43 were diagnosed as iron deficiency anemia (IDA) and 30 as beta-thalassemia trait (BTT) cases. Among the 27 males, 18 were diagnosed as IDA and nine as BTT cases. The mean values were significantly lower in IDA patients for mean corpuscular volume (MCV) (p=.008), mean corpuscular haemoglobin (MCH) (p=.003), and mean corpuscular haemoglobin concentration (MCHC) (p=.003) and significantly higher for red cell distribution width (RDW) (p=.020). The mean ferritin levels in cases of IDA were 7.61 (±3.75) mcg/L and in BTT were 87.09 (±66.77 SD) mcg/L. The mean HbA2 levels in IDA cases were 2.75% (±0.41% SD) and BTT cases were 5.57% (±0.73% SD). CRUISE index revealed the highest AUC (0.934), YI (76.21) and accuracy (90%) followed by the Mentzer index with a diagnostic accuracy of 81%. Shine & Lal index revealed the lowest AUC (0.710), YI (3.28) and accuracy (41%).

Conclusion

CRUISE index, which was recently proposed, was ranked 1^st^ in terms of AUC, YI, and accuracy and was considered 2^nd^ best in terms of sensitivity for differentially diagnosing the two conditions. Mentzer index, a commonly used index, also revealed a high diagnostic accuracy in our study for differentiating BTT from IDA. CRUISE index being a novel index, more research work needs to be carried out in various other geographical setups to evaluate the efficacy of this index.

## Introduction

Anemia is defined as the decrease in red cell mass or blood hemoglobin concentration, ultimately leading to decreased oxygen-carrying capacity of red blood cells, due to which physiological needs of the body are not met. Based on morphology, using mean corpuscular volume (MCV) as an index, anemia can be classified into three major categories, i.e., microcytic anemia (MCV = <80 fl), normocytic anemia (MCV 80-100 fl) and macrocytic anemia (MCV >100 fl) [1]. Anemias that mostly present as microcytic blood picture are: iron deficiency anemia (IDA), thalassemia, sideroblastic anemia and anemia of chronic disorder. Iron deficiency is not only the most common type of microcytic anemia but also the most common form of anemia overall, contributing to approximately 50% of total anemia cases in India. β-thalassemia is the most common form of hemoglobinopathy in India. The overall prevalence of β-thalassemia trait (BTT/βTT) is around 3-4% in India [2]. Though both β-thalassemia trait and iron deficiency anemia (IDA) present with microcytic hypochromic RBCs, their management and prognosis are different. Iron supplementation is used for treating IDA while the same is not recommended in the β-thalassemia trait. In thalassemia patients, misdiagnosed as iron deficiency anemia, iron therapy can be hazardous, as thalassemia is already a hyperferremic condition. So, to avoid inappropriate usage of iron supplements in thalassemia we need to discriminate between thalassemia and iron deficiency anemia. Although hemoglobin-high performance liquid chromatography (Hb HPLC) and serum ferritin levels are considered the gold standard to diagnose beta-thalassemia trait and iron deficiency anemia, respectively, these tests are expensive and are not available to many people especially those living in peripheral areas. Various discrimination indices using mean corpuscular volume (MCV), red blood cell count (RBC count), haemoglobin (Hb), mean corpuscular haemoglobin (MCH), mean corpuscular haemoglobin concentration (MCHC) and red cell distribution width (RDW) have been studied which are not expensive and easily available as a part of complete blood cell count (CBC). Discrimination indices like Mentzer, England and Fraser (E&F), Srivastava, Shine and Lal (S&L), Wongprachum, Kerman I, Kerman II, Green and King (G&K), RDWI, etc. help to differentiate between iron deficiency anemia and thalassemia [3]. Two new indices namely CRUISE index (MCHC + 0.603 RBC + 0.523 RDW) and index26 (a combination of more than 22 indices) have been recently introduced, in the year 2019, and have shown to have a good diagnostic efficacy. Since many studies have not been carried out in our geographical area, it was considered pertinent to analyse the utility of multiple indices in distinguishing between IDA and beta-thalassemia trait in our setup. Through this study, we aim to draw an extensive and comparative analysis of the CRUISE index and other traditional diagnostic indices.

## Materials and methods

This was a laboratory-based observational study conducted in the Central Laboratory, MMIMSR, Ambala, Haryana. A total of 100 cases - over a period of one year and eight months, from August 2022 to March 2024 - were included in this study. All cases with microcytic hypochromic anemia were subjected to CBC analysis and Hb-HPLC testing and were evaluated for serum ferritin. Only cases with MCV < 80 femtoliters (fl) or MCH < 27 picograms (pg) were included in the study. Cases with a history of fever in the last four weeks or who received a blood transfusion or iron therapy in the past 12 weeks were excluded from this study. Cases that had chronic inflammatory conditions, hypothyroidism, acute bleeding episodes or any kind of malignancy were also excluded. Children aged less than 1 year also did not form part of this study group. A total of 5 ml of venous blood was collected using a 22 gauge needle after the puncture site was cleaned using 70% alcohol and thereafter the blood was distributed equally in ethylenediaminetetraacetic acid (EDTA) and plain vials and transported immediately to the laboratory. For CBC parameters, EDTA anticoagulated blood sample was used and was processed within 2 hours of collection using an automated blood cell counter - Sysmex XN-550 (Sysmex Corporation, Kobe, Japan). The CBC parameters that were used for our study were Hb, RBC count, MCV, MCH, MCHC, and RDW-CV. Blood sample collected in the plain vial was used for detecting serum ferritin levels and was then processed using the principle of electrochemiluminescence on a fully automated immunoassay analyser - Cobas e 411. Quality control for ferritin was prepared from a pool of low-concentration defibrinated plasma donations that were repeatedly reactive in the commercial kits. Cases with serum ferritin (less than 15 mcg/l) were considered as iron deficiency anemia cases [4]. For Hb-HPLC, the EDTA vial was stored at 2-8 °C in a refrigerator for a maximum of seven days and HPLC testing was done using D-10 Hemoglobin Testing System (Bio-Rad, Hercules, CA, USA). Cases with HbA2 = 3.5%-3.9% were considered as borderline cases and cases with HbA2 ≥ 4% were considered as β-thalassemia trait cases. A total of eight discrimination indices using various hematological parameters were applied to all the cases included in the study. Indices, along with their formula and cut-off values that were used in the study, have been given in Table 1 [3,5-11].

**Table 1:** Discriminant indices used in our study along with their formulas and cut-off values RBC: red blood cells (million/ul), Hb: hemoglobin (g%), MCV: mean corpuscular volume (fl), MCH: mean corpuscular hemoglobin (pg), MCHC: mean corpuscular hemoglobin concentration (g/dl), RDW: red cell distribution width (%)

SERIAL NO	NAME	FORMULA	CUT-OFF VALUE FOR BTT	CUT-OFF VALUE FOR IDA
1	Mentzer [5]	MCV/RBC	<13	>13
2	Srivastava [6]	MCH/RBC	<3.8	>3.8
3	Shine & Lal [8]	MCV^2 ^× MCH × 0.01.	<1530	>1530
4	Green & King [10]	MCV×MCV×RDW/100Hb	<65	>65
5	RDWI [9]	MCV×RDW/RBC	<220	>220
6	England & Fraser [7]	MCV – RBC – 5Hb – 3.4	<0	>0
7	Kerman I [11]	MCV×MCH/RBC	<300	300-400
8	CRUISE [3]	MCHC + 0.603 RBC + 0.523 RDW	≥ 42.63	<42.63

The data was analysed using SPSS v29.0.2.0 (20) software (IBM Corp., Armonk, NY, USA) and Microsoft Excel (Microsoft® Corp., Redmond, WA, USA). Descriptive statistics were presented as the mean ± standard deviation (SD) and continuous variables conforming to a normal distribution were compared using the independent samples t-test. The sex variable was tested by chi-square test for both the BTT and IDA groups. The Kolmogorov-Smirnov test was used to assess the normality of data distributions. The sensitivity, specificity, positive predictive value, negative predictive value, accuracy, Youden index, and diagnostic odds ratio of each index were calculated according to the previous cut-off value. Receiver operating characteristic (ROC) curves were generated to calculate the areas under the curve (AUC) to determine the cut-off values in our patient population.

## Results

The present study comprised 100 patients with microcytic hypochromic blood picture, out of which 39 cases were of beta-thalassemia trait (BTT) and 61 cases of iron deficiency anemia (IDA). Among the total 100 cases, 73 cases were females and 27 were males. Out of 73 females, 43 were diagnosed as iron deficiency anemia cases and 30 were diagnosed as beta-thalassemia trait cases. Among the 27 males, 18 were diagnosed as iron deficiency anemia cases and nine were diagnosed as beta-thalassemia trait cases. The average age among females was 35.1 (±12.3 SD) years and among male patients was 40.9 (±13.9 SD) years. Among the total 28 cases in the age group of 15-25 years, 14 were BTT cases (five males and nine females) and four were IDA cases (one male and three females). Out of the total 59 cases in the age group of 26-45 years, 20 cases were of BTT (three males and 17 females) and 39 cases were of IDA (eight males and 31 females). In the age group of 46-65 years, two cases were of BTT (both females) and 17 cases were of IDA (nine males and eight females) with a total of 19 cases. In the above 65 years age group, three cases were of BTT (one male and two females) and one female patient of IDA. The statistical analysis of the common CBC parameters is given in Table 2.

**Table 2:** Comparison and analysis of CBC parameters between two groups RBC: red blood cells (million/ul), Hb: hemoglobin (g/dl), MCV: mean corpuscular volume (fl), MCH: mean corpuscular hemoglobin (pg), MCHC: mean corpuscular hemoglobin concentration (g/dl), RDW: red cell distribution width (%), IDA: iron deficiency anemia, BTT: beta-thalassemia trait (p-value <0.05 is considered significant)

	DIAGNOSIS	NUMBER	MEAN	STD. DEVIATION	STD. ERROR MEAN	P-VALUE
Hb (g/dl)	BTT	39	9.477	1.2700	0.2034	0.291
	IDA	61	7.952	1.6989	0.2175	
RBC (million/ul)	BTT	39	5.2218	.71323	0.1142	0.391
	IDA	61	4.1084	.69159	0.0885	
MCV (fl)	BTT	39	65.397	5.16323	0.7734	0.008
	IDA	61	71.884	7.81237	1.0003	
MCH (pg)	BTT	39	19.264	2.24482	0.3595	0.003
	IDA	61	19.5574	3.71346	0.4755	
MCHC (g/dl)	BTT	39	30.528	1.49938	0.2401	0.003
	IDA	61	26.297	2.34954	0.3008	
RDW (%)	BTT	39	18.126	2.94546	0.4649	0.020
	IDA	61	21.4590	4.44175	0.5687	

There was a significant difference in the means of the two groups in parameters like MCV (p=.008), MCH (p=.003), and MCHC (p=.003), which were lower in IDA as compared to BTT. Statistical comparison between the two groups for RDW also revealed a significantly higher mean value in IDA (p=.020) as compared to BTT. The other CBC parameters, i.e. Hb and RBC count, did not show any significant differences in the means of the two groups. The mean ferritin levels in cases of IDA were 7.61 mcg/L with SD of ±3.75 mcg/L whereas mean ferritin levels in cases of BTT were 87.09 mcg/L with SD of ±66.77 mcg/L. The mean HbA2 levels in cases of IDA were 2.75% with SD of ±0.41% whereas the mean HbA2 levels in BTT were 5.57% with SD of ±0.73%. True positive and negative cases (TP and TN), false positive and negative cases (FP and FN) and the total number of correctly identified patients (TP + TN) of each discrimination index for differentiating between BTT and IDA is given in Table 3.

**Table 3:** True positives, False positives, True negatives and False negatives for various indices in our study population TP: true positives, FP: false positives, TN: true negatives, FN: false negatives

DISCRIMINANT INDEX		TP	FP	FN	TN	TP+TN
MENTZER [5]	BTT	24	4	15	57	81
	IDA	57	15	4	24	
SRIVASTAVA [6]	BTT	17	16	22	45	62
	IDA	45	22	16	17	
SHINE & LAL [8]	BTT	39	59	0	2	41
	IDA	2	0	59	39	
GREEN & KING [10]	BTT	11	0	28	61	72
	IDA	61	28	0	11	
RDWI [9]	BTT	16	0	23	61	77
	IDA	61	23	0	16	
ENGLAND & FRASER [7]	BTT	10	0	29	61	71
	IDA	61	29	0	10	
KERMAN I [11]	BTT	30	21	9	40	70
	IDA	40	9	21	30	
CRUISE [3]	BTT	31	2	8	59	90
	IDA	59	8	2	31	

Sensitivity, specificity, positive and negative predictive values (PPV and NPV), accuracy, diagnostic odds ratio (DOR) and Youden index (YI) of each discrimination index for differentiating BTT from IDA with their 95% exact confidence interval are illustrated in Table 4.

**Table 4:** Statistical analysis of various discrimination indices PPV: positive predictive value, NPV: negative predictive value, DOR: diagnostic odds ratio, YI: Youden index

	SENSITIVITY (%)	SPECIFICITY (%)	PPV (%)	NPV (%)	ACCURACY (%)	DOR	YI
MENTZER [5]	61.54% (44.62% to 77.64%)	93.44% (84.05% to 98.18%)	85.71% (69.26% to 94.11%)	79.17% (71.76% to 83.92%)	81%	22.878	54.98
SRIVASTAVA [6]	43.59% (27.81% to 60.38%)	73.77% (60.93% to 84.20%)	51.52% (37.96% to 64.85%)	67.16% (59.91% to 73.68%)	62%	2.173	17.36
SHINE & LAL [8]	100.00% (90.97% to 100.00%)	3.28% (0.40% to 11.35%)	39.80% (38.69% to 40.91%)	100.00% (15.81% to 100.00%)	41%	-	3.28
GREEN & KING [10]	28.21% (15.00% to 44.87%)	100.00% (94.13% to 100.00%)	100.00% (71.51% to 100.00%)	68.54% (64.15% to 72.62%)	72%	-	28.21
RDWI [9]	41.03% (25.57% to 57.90%)	100.00% (94.13% to 100.00%)	100.00% (78.20% to 100.00%)	72.62% (67.12% to 77.51%)	77%	-	38.46
ENGLAND & FRASER [7]	25.64% (13.04% to 42.13%)	100.00% (94.13% to 100.00%)	100.00% (69.15% to 100.00%)	67.78% (63.63% to 71.66%)	71%	-	25.64
KERMAN 1 [11]	76.92% (60.67% to 88.87%)	65.57% (52.31% to 77.27%)	58.82% (49.25% to 67.77%)	81.63% (70.90% to 89.02%)	70%	6.371	39.93
CRUISE [3]	79.49% (63.54% to 90.70%)	96.72% (88.65% to 99.60%)	93.94% (79.71% to 98.39%)	88.06% (79.88% to 93.20%)	90%	114.313	76.21

**Figure 1:**
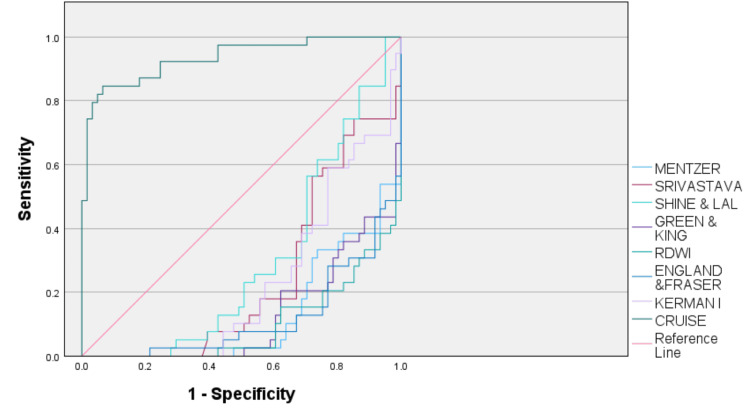
ROC curves of various discriminating indices ROC: receiver operating characteristic curve

Receiver operating characteristic (ROC) curves for all the discrimination indices along with their area under the curve are depicted in Figure 1 and Table 5, respectively.

**Table 5:** Area under curves of various discriminating indices ROC: receiver operating characteristic curve

Area Under the ROC Curve	
Index	Area
MENTZER [5]	0.871
SRIVASTAVA [6]	0.754
SHINE & LAL [8]	0.701
GREEN & KING [10]	0.873
RDWI [9]	0.899
ENGLAND & FRASER [7]	0.881
KERMAN I [11]	0.768
CRUISE [3]	0.934

The proposed cut-off values with optimal sensitivities and specificities for all the discriminating indices pertaining to our study population are given in Table 6.

**Table 6:** The proposed cut-off values of all discriminant indices

INDEX	CUT-OFF VALUE	SENSITIVITY (%)	SPECIFICITY (%)	YOUDEN INDEX
MENTZER [5]	<13.25	93.4%	64.9%	57.5
SRIVASTAVA [6]	<4.44	67.2%	86.6%	51.8
SHINE & LAL [8]	<917.25	68.9%	69.2%	38.1
GREEN & KING [10]	<86.12	98.4%	59%	57.3
RDWI [9]	<259.82	93.4%	69.2%	62.6
ENGLAND & FRASER [7]	<18.98	77.0%	87.2%	64.2
KERMAN I [11]	<303.45	65.6%	79.5%	45.1
CRUISE [3]	>42.14	84.7%	93.4%	78.1

## Discussion

Although beta-thalassemia trait and iron deficiency anemia have different pathogenesis, the clinical presentations in these anemias are quite similar. Moreover, significant differences in their treatment make it vital for discriminating these anemias at an early stage. Normal CBC parameters alone, sometimes are not sufficient to discriminate between these two diseases and therefore a combination of two or more parameters are used leading to significant improvement in discriminant efficacy. Our results revealed that there were significant differences in the means of beta-thalassemia trait patients and iron deficiency anemia patients for parameters like MCV (p=0.008), MCH (p=0.003), MCHC (p=0.003) and RDW (p=0.020). MCV, MCH and MCHC were lower in IDA whereas RDW was significantly higher. No significant difference was seen in the means for parameters like Hb (p=0.291) and RBC count (p=0.391).

There have been a lot of variations in different studies across the globe using single parameters for differentiating BTT from IDA. So, it is important to discriminate between these two disorders along with costly and time-consuming tests; more than 25 discriminating indicators have been proposed in large-scale research and literature for quick and cost-effective differentiation since 1973 to increase the diagnostic efficacy and early treatment of these conditions. Even though a large number of mathematical indices have been proposed for differentiating these two, but none of them have been proven to be 100% sensitive or specific [3]. A considerable variation has been seen in their diagnostic accuracy in different geographical setups. Not many studies had been done in our geographical area, so the present study was conducted using eight discriminant indices, i.e., Mentzer index, Srivastava index, Shine & Lal, Green & King, RDWI, England & Fraser and CRUISE index. CRUISE index, a new index, based on the CRUISE tree algorithm, has recently been proposed for differentiating BTT from IDA [3,12]. In this study, we compared the diagnostic accuracy of all seven older indices along with the newer CRUISE index. Diagnostic accuracy relates to the ability of a test to diagnose a target condition. This discriminative potential of a test or an index can be quantified by the measures of diagnostic accuracy parameters such as sensitivity, specificity, positive predictive value, negative predictive value, likelihood ratios, the area under the ROC curve, Youden's index and diagnostic odds ratio [13]. We have included all the above parameters for a detailed comparison among the different discriminant indices in the present study. These measurements for diagnostic accuracy are very sensitive to the characteristics of a particular population in which the test accuracy is evaluated. So, by interpreting the ROC curves and their area under the curve, diagnostic cut-offs for each index were also calculated for our population. The ROC curve along with the area under the curve (AUC) is a measure to estimate how high is the discriminative power of the test. It represents that the closer the curve is located to the upper left corner, more is the area under the curve and higher is the diagnostic accuracy [13]. A test curve with AUC < 0.5 is not useful for accurately diagnosing a disease state. Test curves with AUC=0.5-0.6, AUC=0.6-0.7, and AUC=0.7-0.8 are considered bad, sufficient, and good, respectively. ROC curve with AUC=0.8-0.9 is considered very good and a curve with AUC=0.9-1 is considered excellent in diagnosing accurately a disease state. A perfect diagnostic test has AUC=1 which practically is very rare especially when a discriminative test with a cut-off value is used to distinguish between two conditions.

Youden’s index is one of the oldest measures for detecting the accuracy of a test [14]. Youden's index is calculated by subtracting 1 from the sum of the test’s sensitivity and specificity and is expressed as a whole number (sensitivity + specificity - 1). So whenever multiple tests are compared for comparing diagnostic accuracy all these measurements may be helpful. In this study, we have ranked all the indices based on parameters like sensitivity, specificity, PPV, NPV, AUC, accuracy and Youden’s index. The ranking of diagnostic performance of discrimination indices for differentiating BTT from IDA in patients with microcytic anemia is depicted in Table 7.

**Table 7:** Ranks of various indices for diagnostic parameters (lower rank means higher diagnostic potential) TPR: true positive rate, TNR: true negative rate, FPR: false positive rate, PPV: positive predictive value, NPV: negative predictive value, AUC: area under curve, YI: Youden index (Top 4 ranks have been bolded)

INDEX	TPR	TNR	PPV	NPV	Accuracy	AUC	YI
MENTZER [5]	4	5	5	4	2	4	2
SRIVASTAVA [6]	5	6	7	8	7	7	7
SHINE & LAL [8]	1	8	8	1	8	8	8
GREEN & KING [10]	7	1	1	6	4	5	5
RDWI [9]	6	1	1	5	3	2	4
ENGLAND & FRASER [7]	8	1	1	7	5	3	6
KERMAN I [11]	3	7	6	3	6	6	3
CRUISE [3]	2	4	4	2	1	1	1

In our study, the highest diagnostic sensitivity was shown by Shine & Lal index (100%) which is in concordance with studies conducted by Huang et al., Okan et al., Khan et al. and Jahangiri et al. [3,15-17]. CRUISE index, Kerman I and Mentzer also showed good diagnostic sensitivity having 79.49%, 76.92%, and 61.54% sensitivities, respectively. England & Fraser index proved to be the least sensitive with around 26.64% sensitivity. Green & King, RDWI and England & Fraser all had a specificity of 100% followed by CRUISE index (96.72%) and Mentzer index (93.44%). Okan et al. and Huang et al. in their respective studies also revealed a comparable specificity of all three indices [15,17]. Our study revealed Shine & Lal index to be the least specific with a specificity of 3.28% which is concordant with the study conducted by Jahangiri et al. in Iran, in which Shine & Lal had a specificity of just 17.57%, the lowest out of all the 26 indices included in their study. PPV was also highest for Green & King, RDWI and England & Fraser with each having 100% positive predictive value. CRUISE index and Mentzer also revealed high positive predictive values having 96.72% and 93.44%, respectively. The index with least PPV was Shine & Lal having 39.8% positive predictive value which was also the case in Jahangiri’s study in which Shine & Lal had a PPV of 63.87%, 3rd lowest among all 26 indices used [4]. NPV was highest in the Shine & Lal index (100%) and lowest in the Srivastava index (67.16%) which was also seen by Jahangiri et al. in their study [3].

The highest diagnostic accuracy for discriminating BTT from IDA was shown by the newly proposed index i.e., CRUISE index with an accuracy of 90%, followed by Mentzer index (81%), RDWI (77%) and Green & King (72%). Shine & Lal revealed the lowest diagnostic accuracy with diagnosing only 41% of cases correctly. The accuracy was similar for the Mentzer index and Shine & Lal index in Jahangiri’s study with Mentzer being 3rd most accurate in distinguishing beta-thalassemia trait from iron deficiency anemia [3].

ROC curves depicting the area under the curves revealed a value of 0.934 for the CRUISE index, the highest in our study. The 2nd highest area under the curve was shown by RDWI with an approximate value of 0.914. These two tests revealed an AUC of greater than 0.9 which makes them an ‘excellent’ discriminant test for distinguishing beta-thalassemia trait from iron deficiency anemia according to the standard protocols. England & Fraser, Mentzer, and Green & King revealed an AUC of 0.893, 0.884, 0.883 respectively making them a ‘very good’ discriminant index. Kerman I and Srivastava index had AUC equalling 0.775 and 0.758 respectively, making them a ‘good’ discriminant test. Shine & Lal revealed an AUC of 0.710, the least in our study population.

The Youden index is an old and efficient way to summarise the diagnostic efficacy of a test. In our study population, the CRUISE index revealed a YI=76, the highest among all eight indices, followed by the Mentzer index (YI=54), Kerman I (YI=39), RDWI (YI=38), Green & King (YI=28), England & Fraser (YI=25), and Srivastava (YI=17). The discriminant index with the least value was Shine & Lal, having YI=3, as the cut-off values for discriminant tests vary in different clinical and geographical setups. Therefore, a proposed cut-off value for all eight indices was also evaluated for our study population using ROC curves along with their AUC. A comparison is drawn between the standard cut-off values and the proposed cut-off values for all the indices in patients of our geographical area in Table 8.

**Table 8:** Comparison of standard and proposed cut-offs of discriminant indices for our patient population

INDEX	STANDARD CUT-OFF	PROPOSED CUT-OFF
MENTZER [5]	<13	<13.25
SRIVASTAVA [6]	<3.8	<4.44
SHINE & LAL [8]	<1530	<917.25
GREEN & KING [10]	<65	<86.12
RDWI [9]	<220	<259.82
ENGLAND & FRASER [7]	<0	<18.98
KERMAN I [11]	<300	<303.45
CRUISE [3]	>42.63	>42.14

It was also seen that the proposed cut-off values for our patient population in all the discriminant indices were comparable to other studies. Xiao et al. in their study proposed a similar cut-off value for the Srivastava index i.e., 4.56 with our cut-off value being 4.44 for distinguishing BTT from IDA [18]. Similarly, for the Shine & Lal index, the cut-off value proposed by Xiao et al. was much lower than the standard cut-off value with a value of 1187, which was also the case in our study. Also, for other indices like Green & King and RDWI, Xiao et al. proposed a revised cut-off value of 82.85 and 249.48, respectively, which was close to our proposed cut-off value of 86.12 and 259.82 for G&K and RDWI, respectively. Kumar et al. in their study conducted in 2017 proposed a much higher cut-off value of 11.06 for the England & Fraser index as compared to the standard cut-off value [19]. Even our study revealed a similar result in which the proposed cut-off for England and Fraser (E&F) was 18.98, much higher than the standard cut-off value of 0. It was also seen that the cut-off values for the Mentzer index (13.25) and Kerman I (303.45), as proposed in our study for distinguishing BTT from IDA, were almost identical to the standard cut-off values of 13 and 300, respectively.

The current study was conducted on a relatively small sample size of 100 cases from a single geographical area in North India, which limits the generalizability of the findings and was a major limitation in this study. Also, the study included a newly introduced index for which further validation across different populations and larger sample sizes is required to establish its reliability and diagnostic accuracy. The study also did not include cases with borderline HbA2 levels. The study of such cases is likely to provide more insights into the differential diagnosis of beta-thalassemia trait and iron deficiency anemia.

## Conclusions

By using all the diagnostic accuracy parameters, a detailed comparison was drawn out among all the eight indices reflecting their discriminant potential to diagnose beta-thalassemia trait and iron deficiency anemia correctly. In our study population CRUISE index, based on the CRUISE tree algorithm, demonstrated superior diagnostic accuracy to other indices studied. As it is a new index more research needs to be carried out in various other geographical setups to evaluate the efficacy of this index. With the Mentzer index, RDWI, Green & King and other indices already in use by clinicians in their daily practice, the CRUISE index should also be considered when differentiating the BTT from IDA. The study also proposed specific cut-off values for each discrimination index for the study population, which can provide practical guidelines for accurate diagnosis of BTT and IDA in our geographical area.
